# Interpersonal Problems and Their Impact on Alliance Ruptures in Psychotherapy

**DOI:** 10.1002/cpp.70071

**Published:** 2025-04-02

**Authors:** Flavio Iovoli, Marie Baranowski, Leonie S. Sander, Julian A. Rubel

**Affiliations:** ^1^ Department of Psychology Osnabrueck University Osnabrueck Germany

**Keywords:** Alliance ruptures, cognitive–behavioural therapy, confrontation, interpersonal problems, therapeutic alliance, withdrawal

## Abstract

**Introduction:**

Alliance ruptures are frequent occurrences in psychotherapy. Understanding the factors that contribute to these ruptures is important for optimizing therapeutic outcomes. From the perspective of interpersonal theory, the experience of interpersonal problems may play a significant role in the occurrence of alliance ruptures, as maladaptive relational patterns can trigger momentary interpersonal misattunements. This study aimed to investigate how specific interpersonal problems predict the frequency of alliance ruptures in the first three sessions of cognitive–behavioural therapy.

**Methods:**

Sixty‐four patients completed the Inventory of Interpersonal Problems (32 items) (IIP‐32) as part of their routine outcome monitoring in an outpatient clinic. Subsequently, the first three regular therapy sessions were coded using the Rupture and Resolution Rating System (3RS). Multilevel Bayesian hurdle models were estimated, simultaneously modelling (1) the likelihood of experiencing at least one rupture and (2) the frequency of rupture occurrences among patients who had at least one.

**Results:**

Withdrawal ruptures were more frequent than confrontation ruptures. Higher affiliation and friendly–submissive tendencies were associated with fewer withdrawal ruptures but increased the likelihood of experiencing at least one withdrawal rupture. In contrast, hostile–submissive tendencies predicted more frequent withdrawal ruptures but were associated with a lower probability of at least one occurrence. For confrontation ruptures, the overall level of dysfunction and self‐sacrificing tendencies predicted fewer confrontation ruptures.

**Conclusion:**

These findings suggest that specific interpersonal problems can provide therapists with crucial insights into subtle rupture occurrences. By understanding these dynamics, therapists can better recognize and address ruptures, ultimately enhancing the therapeutic process.

**Summary**

- Alliance ruptures are common in psychotherapy.
- Interpersonal problems may increase the likelihood of alliance ruptures, making them valuable areas for therapists to monitor.
- This study found that hostile submissive tendencies predicted higher frequencies of withdrawal ruptures, while friendly submissive tendencies were associated with fewer withdrawal ruptures.
- Confrontation ruptures were less frequent and less systematically related to patient‐reported interpersonal dysfunction.
- Understanding patients' interpersonal styles can help therapists identify subtle ruptures early, enabling timely interventions and strengthening the therapeutic alliance.

## 1 Introduction

In its traditional form, psychotherapy is inherently interpersonal where a patient and their therapist interact and build a therapeutic alliance over numerous sessions in order to attain their therapy goals (Orlinsky and Howard 1987). This interaction is influenced not only by the therapeutic techniques but also by the relational patterns that both the patient and the therapist bring into the therapeutic context (Hatcher 2015; Strong 1968). Accordingly, Bordin (1979) described the therapeutic alliance as a *function of fit* between the personalities of parts of the dyad. Therefore, in understanding the therapeutic alliance, it becomes important to consider the patterns of interaction that unfold between the patient and therapist (Zilcha‐Mano and Muran 2024). One useful framework for conceptualizing these patterns is the interpersonal theory influenced by Sullivan (1953) and Leary (1957). According to interpersonal theory, individuals tend to elicit specific responses based on their interpersonal behaviour along two primary dimensions: affiliation (ranging from hostile to friendly) and dominance (ranging from dominant to submissive; Leary 1957). For example, dominant–hostile behaviour is thought to invite a submissive–hostile response (Horowitz 1996). In the psychotherapeutic context, these complementary behaviours can significantly influence the therapeutic alliance. For example, Kiesler and Watkins (1989) found that interpersonal complementarity in the hostile hemisphere was associated with a stronger early alliance. They hypothesized that therapists may initially respond in ways that align with the patient's interpersonal style, which can facilitate alliance formation in the early stages of therapy. However, when hostility was too extreme, patients and therapists both perceived the alliance as weaker (Kiesler and Watkins 1989).

Patients frequently report the experience of distress stemming from difficulties in the interpersonal domain, which often manifest as challenges in navigating social interactions effectively (Girard et al. 2017; Horowitz 1979). One of the most widely used instrument to assess these difficulties is the *Inventory of Interpersonal Problems* (IIP; Horowitz et al. 1988). The IIP conceptualizes interpersonal problems as maladaptive behaviour that can be categorized into excesses (doing something too much) and inhibitions (it is hard to do something; Horowitz 1979). In addition to a general indicator reflecting overall interpersonal distress, the IIP identifies eight subtypes of interpersonal problems, organized into a circumplex model (Alden, Wiggins, and Pincus 1990), reflecting variations along the two primary axes of affiliation and dominance (Horowitz et al. 2000; Wiggins 1996). The domineering subscale (PA) captures issues related to being overly controlling and aggressive in social interactions, whereas self‐centred (bc) individuals tend to prioritize their own needs over those of others. The cold subscale (DE) reflects difficulties in forming close relationships and expressing emotions, while socially inhibited (FG) individuals experience problems in joining groups, making friends and open themselves up. Items of the non‐assertive subscale (HI) assess the individual's ability to assert their own needs, setting boundaries and being confident. Overly accommodating (JK) individuals are overly compliant, rarely saying ‘no’ to others. The self‐sacrificing (LM) subtype experiences themselves as wanting to please others too much, are overly trusting and being too influenced by others. Lastly, intrusive (NO) individuals have problems in maintaining appropriate boundaries and have a tendency to become overly involved in others' affairs (Ruiz et al. 2004; Thomas, Brähler, and Strauß 2011). Beyond these specific subtypes, Gurtman (1996) introduced the grouping of interpersonal problems into four broader quadrants within the circumplex model: friendly–dominant (FD), friendly–submissive (FS), hostile–dominant (HD) and hostile–submissive (HS). These quadrants are formed by combining adjacent octants, capturing broader patterns of interpersonal tendencies and relational difficulties.

Interpersonal problems are strongly linked to various forms of mental health impairments. Higher levels of interpersonal problems are related to more severe symptoms of depression (Ansell, Grilo, and White 2012; Koppelberg, Kersting, and Suslow 2023; Schmitz et al. 2000), higher levels of anxiety symptoms (Akyunus and Gencoz 2016; Dally et al. 2005; Kim and Bae 2022), less positive and more negative emotions (Liu et al. 2024; Prout et al. 2021; Ringwald, Woods, and Wright 2024) and higher levels of perceived stress (Rakhimov et al. 2023). Given these widespread associations, interpersonal problems are likely to be a core psychopathological characteristic (Girard et al. 2017; Hopwood et al. 2013; McEvoy et al. 2013). Indeed, patients seeking psychotherapy frequently report interpersonal difficulties, underscoring their prevalence in therapeutic settings (Horowitz 1979). However, these interpersonal problems serve as predictors of poorer therapeutic outcomes (Gómez Penedo and Flückiger 2023). Furthermore, interpersonal problems do not only predict poorer therapeutic outcomes but also influence the therapeutic process itself. A recent longitudinal study found that higher‐than‐usual levels of interpersonal problems within a session were linked to a weaker‐than‐usual perception of the alliance in the same session and even five sessions later (Iovoli et al. 2025). These findings align with a recent meta‐analysis showing that patients with more pronounced interpersonal problems at the beginning of psychotherapy are at risk for developing weaker therapeutic alliances, especially if the alliance was assessed in the first third of the treatment (Iovoli et al. 2024). That said, the research was more ambiguous, with some studies reporting positive correlations between interpersonal problems and subsequent therapeutic alliance. In these cases, rather than undermining the therapeutic alliance, interpersonal difficulties seemed to serve as a resource that strengthened the bond between therapist and patient. More specifically, findings suggest that not all interpersonal problems impact the perception of the therapeutic alliance in the same (or even consistent) way. For instance, patients with friendly–submissive dispositions (e.g., non‐assertive or overly accommodating) tend to establish stronger early alliances (Hardy et al. 2001; Muran et al. 1994). Further evidence suggests that improvements in the therapeutic alliance within a given patient–therapist dyad may be particularly therapeutic for patients with high affiliation and low dominance, as alliance strengthening over time has been linked to greater symptom reduction in this group. Conversely, for patients with lower levels of affiliation and higher dominance, changes in the alliance appeared to be less predictive of symptom improvement, suggesting that the therapeutic relationship itself plays a more central role in treatment outcomes for overly accommodating patients (Constantino et al. 2023). In contrast, hostile–dominant interpersonal styles (e.g., cold, distant or self‐centred behaviours) consistently predict weaker alliances (Hersoug et al. 2002, 2009; Krieg and Tracey 2016), potentially due to difficulties in goal agreement (Muran et al. 1994). This pattern suggests that high affiliation facilitates alliance formation, while low affiliation and high dominance may create barriers to collaboration. Because the therapeutic alliance is shaped by patients' interpersonal tendencies, difficulties in collaboration may arise when these styles create misattunements or tensions between patient and therapist (Iovoli et al. 2024).

This proposal aligns with the theory of alliance ruptures and repairs, which provides a more dynamic view on the therapeutic alliance (Eubanks, Samstag, and Muran 2023; Safran and Muran 1996). According to this approach, the therapeutic alliance is subject to fluctuations, marked by moments of tension or breakdowns, referred to as alliance ruptures (Safran et al. 2001). These ruptures *disrupt* the collaborative relationship, which is built on three key components: agreement on therapeutic goals, agreement on tasks and the emotional bond between patient and therapist (Bordin 1979; Eubanks, Samstag, and Muran 2023). Ruptures can manifest as acts of withdrawal, where either the patient or the therapist distance themselves from the dyad by becoming less responsive or engaging in abstract communication, or as acts of confrontation, where they express criticism or hostility toward the other (Eubanks et al. 2019; Eubanks and Muran 2023).

Ruptures often arise from momentary interpersonal misattunement, where one part of the therapeutic dyad fails to adequately respond to the other's needs (Safran and Kraus 2014). Research consistently shows that unresolved tensions in the alliance make the therapeutic process more difficult and less effective (Coutinho et al. 2011). When left unresolved, these ruptures can weaken the alliance and have been shown to predict poorer therapeutic outcomes (Babl et al. 2022; Rubel et al. 2018). However, the rupture–repair model emphasizes that these moments do not necessarily indicate a failing intervention (Safran et al. 2001). On the contrary, when ruptures are identified and addressed, repairs occur, which can ultimately strengthen the alliance (Eubanks, Samstag, and Muran 2023; Eubanks, Muran, and Safran 2019). Successfully repaired ruptures not only restore the collaborative relationship but often lead to a more resilient bond between patient and therapist (Eubanks, Burckell, and Goldfried 2018). Studies have demonstrated that the repair of ruptures is associated with stronger therapeutic alliances, better treatment outcomes, symptom reduction and improved psychological functioning (Eubanks, Muran, and Safran 2018; Muran et al. 2009; Zilcha‐Mano and Errázuriz 2017).

Alliance ruptures are therefore an important in‐session process, as they offer key opportunities for relational growth when handled effectively (Eubanks, Samstag, and Muran 2023). In addition, alliance ruptures are frequent occurrences in psychotherapy (Cirasola et al. 2024; Muran and Eubanks 2020). Hence, it is important to consider what factors might influence the occurrence of ruptures in psychotherapy (Castonguay et al. 2010). Given their central role in shaping therapeutic interactions as outlined earlier, interpersonal problems may be particularly relevant for understanding and predicting rupture processes (Gómez Penedo et al. 2024; Høgenhaug, Kongerslev, and Kjaersdam Telléus 2024). Specifically, Zilcha‐Mano and Muran (2024) highlight the importance of assessing patients' interpersonal tendencies at the outset of therapy to guide rupture–repair strategies, noting that the frequency of alliance ruptures itself may be an informative marker for individualizing treatment approaches. Supporting this, Luo et al. (2021) provided initial evidence that different interpersonal tendencies may contribute to distinct rupture types. Using observer‐rated in‐session behaviours, they found that dominant and cold interpersonal behaviours were linked to confrontation ruptures, while withdrawal ruptures were associated with interpersonal warmth.

While alliance ruptures are a frequent and meaningful process in psychotherapy, the factors influencing their occurrence remain insufficiently understood. Given that interpersonal tendencies shape how patients engage in relationships, they likely play a role in the subtle, often unspoken tensions that manifest as alliance ruptures (Høgenhaug, Kongerslev, and Kjaersdam Telléus 2024; Zilcha‐Mano and Muran 2024). However, despite the well‐established impact of interpersonal problems on therapeutic interactions, little research has examined their role in predicting rupture frequency over the course of therapy. Identifying such patterns could be particularly valuable for treatment planning, as therapists who are aware of a patient's interpersonal tendencies may be more attuned to subtle ruptures and better equipped to address them in a timely manner. To address this gap, the present study examines how interpersonal problems, as assessed with the IIP, predict the frequency of withdrawal and confrontation ruptures in the early phase of cognitive–behavioural therapy. The IIP remains the most widely used and validated measure for capturing interpersonal difficulties, making it a valuable tool for investigating how dispositional interpersonal patterns contribute to alliance ruptures. Specifically, we test this at four levels of analysis: (1) overall interpersonal dysfunction, (2) affiliation and dominance, (3) quadrant scores and (4) octant scores. Comparing these levels allows for identifying which level of the interpersonal dysfunction is most informative for predicting alliance ruptures, with finer distinctions at the quadrant and octant levels potentially offering greater clinical utility for case conceptualization and treatment planning (Zilcha‐Mano and Muran 2024).

At the overall level of interpersonal dysfunction, higher interpersonal distress is expected to predict a greater frequency of both withdrawal and confrontation ruptures, as more severe interpersonal difficulties likely contribute to heightened relational strain, misattunements and disruptions in collaboration within the therapeutic dyad (H1; Iovoli et al. 2024, 2025). At the level of interpersonal dimensions (affiliation and dominance), higher affiliation is expected to predict fewer withdrawal ruptures (H2), while higher dominance is expected to predict more confrontation ruptures (H3). In addition to affiliation and dominance, we examine quadrants and octants, as these finer distinctions may offer greater clinical utility for practitioners. At the quadrant level, higher hostile–dominant (HD) tendencies are expected to predict more confrontation ruptures (H4), while higher hostile–submissive (HS) tendencies are expected to predict more withdrawal ruptures (H5). Lastly, an exploratory analysis will examine octants to determine whether specific interpersonal subtypes within the quadrants play a particularly salient role in predicting withdrawal and confrontation frequency.

## 2 Method

### 2.1 Sample

The sample in the present study consisted of 64 patients undergoing psychotherapeutic treatment in an outpatient setting for adults. The patients were on average 35.36 years old (*SD* = 15.86, range = 18–77) and the majority (67.2%) identified as female. Around half of the patients (46.9%) were primarily diagnosed with an affective disorder, most prominently the recurrent depressive disorder with currently a moderate episode. Other prevalent primary diagnoses were some sort of anxiety disorder (25%), adjustment disorder (7.8%) and personality disorder (7.8%); 54.7% of the patients had at least one comorbid disorder. The majority of patients were unmarried (57.8%), in a committed relationship (64.0%), had some sort of higher education (57.8%) and were employed at the time of assessment (57.8%).

### 2.2 Treatment

Patients received disorder‐oriented cognitive–behavioural therapy (Margraf and Schneider 2008) in the outpatient psychotherapy clinic Osnabrueck University, Northwestern of Germany. Exclusion criteria from the outpatient clinic were acute suicidality, acute violence, acute organic mental disorder, acute psychotic disorder or acute disorder due to psychotropic substances. All treatments were conducted by trainee therapists in advanced psychotherapy training program as part of their practical training. Trainees were supervised by an experienced licensed psychotherapist every fourth session. During the probatory phase, patients were assessed using psychometric assessments and a structured clinical interview (SKID‐I, Wittchen 1997). Additionally, they provided informed consent. In Germany, the probatory phase consists of up to six sessions, which allows the patient and therapist to determine if they are a good match for working together, and whether the therapeutic approach being offered is suitable for the patient's need. During this phase, an application for insurance coverage is submitted. Once approval is granted, the regular course of therapy begins. In the present sample, the patients were treated by 25 trainees, who were mostly female (79.7%).

### 2.3 Measurements

#### 2.3.1 Alliance Ruptures

The frequency of alliance ruptures was assessed using the Rupture and Resolution Rating System (3RS v2022; Eubanks and Muran 2023). The 3RS is an observer‐based rating system, where trained coders rate the dyad on working‐together quality, ruptures (withdrawal and confrontation) and repair efforts in five‐minute segments (Eubanks, Muran, and Safran 2014). Previously, it has been shown to be a reliable coding system (Eubanks et al. 2019). The coding team consisted of four graduate students who were trained by the first author. The training comprised three sessions, each lasting 3 h. The first session focused on the therapeutic alliance and the rupture and resolution theory, during which the coders were introduced to the 3RS. In the second session, the team watched and coded videos of Gloria with Fritz Perls and Carl Rogers, as these examples are utilized by the developers of the 3RS. In the final session, a video from the outpatient clinic was coded collectively, with each segment being discussed in detail in terms of ruptures, repairs and collaborative work. In the 3RS, alliance ruptures are evaluated on a 5‐point Likert scale, ranging from 1 for no rupture to 5 very significant ruptures in this segment. In the 3RS, a score of 3 represents a clear, ‘textbook’ rupture. However, for the purpose of our study, we were particularly interested in the frequency of alliance ruptures. The criterion variable was therefore the total number of rupture instances observed for both patient and therapist, combined into a single indicator representing the overall rupture frequency within the dyad. Given that a score of 3 inherently implies a higher intensity, we decided to use a score of 2 as a dichotomous indicator of a rupture. Consequently, the frequency of alliance ruptures was determined by the presence of scores equal to or greater than 2 within the first three sessions. While this approach does not capture the precise number of discrete rupture events, it serves as a proxy for rupture frequency, as the coding system does not quantify the actual amount of multiple ruptures within a single segment. The interrater‐reliability was ICC(3,1) = 0.72 for the dichotomous rating (rupture yes or no), which constitutes substantial to good agreement across raters (Shrout and Fleiss 1979).

#### 2.3.2 Types of Interpersonal Problems

Interpersonal problems were assessed using the 32‐item German version of the Inventory of Interpersonal Problems (IIP‐32; Thomas, Brähler, and Strauß 2011). The IIP is a self‐report instrument, where individuals rate their interpersonal inhibitions and excesses on a 5‐point Likert scale, with responses ranging from 0 (not at all) to 4 (extremely). The scale includes the following subscales (octants) that measure specific interpersonal problem types, which are combinations of the dimensions of affiliation and dominance: domineering, intrusive, overly nurturant, overly accommodating, non‐assertive, socially avoidant, cold and vindictive (Horowitz et al. 2000; Thomas, Brähler, and Strauß 2011). To calculate the subscales, the unipsatized scores were used as it removes the general distress information (Becker and Mohr 2005; Grosse Holtforth, Lutz, and Grawe 2006; Zimmermann and Wright 2017). As recommended, the subscales were z‐standardized (Thomas, Brähler, and Strauß 2011). Additionally, the quadrant scores of friendly–dominant (FD), friendly–submissive (FS), hostile–dominant (HD) and hostile–submissive (HS) can be calculated using the formula provided by Ruiz et al. (2004): FD = intrusive + (0.707 × domineering) + (0.707 × self‐sacrificing); FS = overly accommodating + (0.707 × overly nurturant) + (0.707 × non‐assertive); HD = socially inhibited + (0.707 × domineering) + (0.707 × cold); and HS = socially inhibited + (0.707 × non‐assertive) + (0.707 × cold). In the present study, the reliability was Cronbach's *α* = 0.84.

### 2.4 Procedure

Patients completed the IIP‐32 as part of their intake assessment. After the probatory phase was completed and the therapy was approved, patients commenced their treatment, which was videotaped in its entirety. Following the initiation of the treatment, the coders rated the first three regular therapy sessions for each patient using the 3RS. The decision to focus on this specific time phase was based on several considerations. First, we excluded the probatory phase from coding because it is highly standardized and structured due to the use of the structured clinical interview. Additionally, the first therapy session after the approval is often exploratory or involves discussion about the break due to the approval process. By analysing the three subsequent sessions, we aimed to capture both the early stages of therapy, and a phase where some therapeutic techniques were actively applied.

### 2.5 Data Analysis

To examine the relationship between interpersonal problems and the frequency of alliance ruptures within the dyad, a multilevel Bayesian hurdle Poisson model was used (Mullahy 1986). A Bayesian approach was chosen because it handles small sample sizes more robustly (McNeish 2016), explicitly models parameter uncertainty and allows for the incorporation of weakly informative priors, preventing overfitting while maintaining flexibility in estimation (Gelman et al. 2008). This modelling approach was necessary because zero values represent a qualitatively different process than non‐zero values. Specifically, the step from 0 to 1 indicates the occurrence of a rupture, while increases from 1 to 2 or higher simply reflect the frequency of ruptures once they have occurred. Specifically, the hurdle model consists of two components: (1) a logistic regression predicting the probability of any rupture occurring (zero vs. non‐zero) and (2) a Poisson regression modelling the frequency of ruptures among those who experienced at least one. The models were implemented using the *R* package *brms* with 10,000 iterations and a seed of 123 (Bürkner 2017).

Given that rupture frequencies are nested within therapists, a two‐level structure was implemented, with 64 patients at level 1 and 25 therapists at level 2. Intraclass correlation coefficients (ICCs; Raudenbush and Bryk 2002) were calculated to assess the proportion of variance attributable to therapists. For non‐Gaussian data, ICCs were approximated using the delta method, specifically using for the residual variance the formula log(1 + (1/λ)), where λ represents the mean rupture frequency (Nakagawa, Johnson, and Schielzeth 2017).

To evaluate model performance and compare different levels of interpersonal predictors (overall dysfunction, interpersonal dimensions, quadrants and octants), models were assessed using leave‐one‐out cross‐validation (LOO‐CV; Vehtari, Gelman, and Gabry 2017), which was implemented in *R* with the package *loo* (Vehtari et al. 2015) To evaluate model performance, we used the Expected Log Predictive Density (ELPD), where higher values indicate better predictive accuracy.

All analyses were conducted in *R* (version 4.4.2, R Core Team 2018).

## 3 Results

### 3.1 Descriptives

The frequency of withdrawal ruptures was analysed using the first three therapy sessions for each dyad. Across the sample, the mean frequency of withdrawal ruptures was 4.45 (*SD* = 4.72, range = 0–28). When examined per session, the mean frequency was 1.78 (*SD* = 2.39, range = 0–11) in Session 1, 1.34 (*SD* = 1.73, range = 0–9) in Session 2 and 1.33 (*SD* = 1.8, range = 0–9) in Session 3. The mean frequency of confrontation ruptures was 1.69 (*SD* = 2.32, range = 0–10). For each session, the mean frequency was 0.86 (*SD* = 1.49, range = 0–7) in Session 1, 0.47 (*SD* = 1.04, range = 0–4) in Session 2 and 0.36 (*SD* = 0.68, range = 0–3) in Session 3. Table 1 presents the descriptive statistics for interpersonal problem variables alongside their Spearman correlations with withdrawal and confrontation rupture frequency.

**TABLE 1:** Descriptives and correlations between interpersonal problems and rupture frequency.

	Descriptives		Correlations			
			Withdrawal ruptures		Confrontation ruptures	
	Mean	SD	ρ	*p*‐Value	ρ	*p*‐Value
IIP mean	1.60	0.44	0.002	0.988	−0.161	0.203
Affiliation	−0.03	0.35	−0.293	0.012	0.139	0.275
Dominance	−0.18	0.41	−0.205	0.105	0.241	0.055
Friendly–Dominant	3.39	1.33	−0.102	0.424	0.013	0.916
Friendly–Submissive	4.18	1.23	−0.108	0.395	−0.167	0.186
Hostile–Dominant	3.74	0.96	0.004	0.972	−0.084	0.510
Hostile–Submissive	3.98	1.67	0.210	0.095	−0.269	0.031
Domineering (PA)	1.68	0.85	0.082	0.522	0.174	0.168
Self‐centred (bc)	1.60	0.50	−0.143	0.258	−0.166	0.189
Cold (DE)	1.34	0.72	0.100	0.431	−0.270	0.031
Socially inhibited (FG)	1.89	0.87	0.216	0.087	−0.220	0.081
Non‐assertive (HI)	1.61	0.74	0.232	0.066	−0.280	0.025
Overly accommodating (JK)	2.07	0.68	−0.218	0.084	−0.123	0.331
Self‐sacrificing (LM)	1.38	0.73	−0.125	0.324	−0.094	0.462
Intrusive (NO)	1.23	0.55	−0.163	0.198	−0.139	0.273

Abbreviations: SD, standard deviation; IIP, Inventory of Interpersonal Problems.

### 3.2 Prediction of Withdrawal Rupture Frequency

The analysis revealed that interpersonal problems significantly predicted the frequency of withdrawal ruptures (see Table 2). Among the four models tested, the interpersonal quadrant model provided the best predictive performance (Δ ELPD = 0.0, SE = 0.0), followed by the octant model (Δ ELPD = −7.1, SE = 9.5), the interpersonal dimension model (Δ ELPD = −5.1, SE = 11.9) and the overall interpersonal dysfunction model, which performed the worst (Δ ELPD = −12.2, SE = 16.4). The ICCs indicated that a substantial proportion of variance in withdrawal rupture frequency was attributable to therapists, ranging from 53.8% to 66.97% across models. The high ICC values suggest that withdrawal ruptures are not merely patient‐driven but arise from the therapist–patient dyad, indicating that some dyads systematically experience more ruptures than others.

**TABLE 2:** Prediction of withdrawal rupture frequency.

	Model parameters		ICC	Model comparison	
Predictors of the models	Estimate (estimate error)	95% CI		Δ ELPD	SE (Δ ELPD)
Overall interpersonal dysfunction			65.16%	−12.2	16.4
IIP mean	−0.1213 (0.1219)	[−0.3619, 0.1160]			
Interpersonal dimensions			66.97%	−5.1	11.9
Affiliation	**−0.4225 (0.2081)**	**[−0.8373, −0.0192]**			
Dominance	−0.1549 (0.1728)	[−0.4939, 0.1844]			
Interpersonal quadrants			61.44%	0.0	0.0
Friendly–Dominant	0.0854 (0.0583)	[−0.0300, 0.1978]			
Friendly–Submissive	**−0.2076 (0.0726)**	**[−0.3507, −0.0660]**			
Hostile–Dominant	−0.1588 (0.0836)	[−0.3230, 0.0057]			
Hostile–Submissive	**0.2549 (0.0552)**	**[0.1478, 0.3642]**			
Interpersonal octants			53.80%	−7.1	9.5
Domineering (PA)	0.0388 (0.0919)	[−0.1447, 0.2177]			
Self‐centred (bc)	−0.1299 (0.0904)	[−0.3061, 0.0480]			
Cold (DE)	**−0.3336 (0.1390)**	**[−0.6068, −0.0620]**			
Socially inhibited (FG)	**0.3597 (0.1078)**	**[0.1508, 0.5738]**			
Non‐assertive (HI)	**0.3793 (0.1430)**	**[0.0932, 0.6559]**			
Overly accommodating (JK)	**−0.3841 (0.1118)**	**[−0.6023, −0.1634]**			
Self‐sacrificing (LM)	0.0308 (0.1305)	[−0.2318, 0.2815]			
Intrusive (NO)	0.0421 (0.1319)	[−0.2149, 0. 3021]			

*Note:* Values in boldface highlight estimates with a credible departure from the null value.

Abbreviations: ICC, intraclass correlation coefficient; IIP, Inventory of Interpersonal Problems; SE, standard error; Δ ELPD, difference in Expected Log Predictive Density between models.

Higher affiliation was associated with fewer occurrences of withdrawal ruptures (*β* = −0.423, 95% CI [−0.837, −0.019]). At the quadrant level, higher hostile–submissive tendencies significantly predicted more withdrawal ruptures (*β* = 0.255, 95% CI [0.148, 0.364]), whereas higher friendly–submissive tendencies were associated with fewer withdrawal ruptures (*β* = −0.208, 95% CI [−0.351, −0.066]). At the octant level, socially inhibited (*β* = 0.360, 95% CI [0.151, 0.574]) and non‐assertive tendencies (*β* = 0.379, 95% CI [0.093, 0.656]) were associated with higher withdrawal rupture frequency, while overly accommodating (*β* = −0.384, 95% CI [−0.602, −0.163]) and cold (*β* = −0.334, 95% CI [−0.607, −0.062]) tendencies predicted lower withdrawal ruptures.

In addition to predicting rupture frequency, some variables significantly predicted the likelihood of experiencing at least one withdrawal rupture (hurdle component). Higher affiliation (95% CI [0.15, 7.16]) and friendly–submissive tendencies (95% CI [0.03, 3.54]) were associated with an increased probability of experiencing at least one withdrawal rupture, while higher hostile–submissive tendencies (95% CI [−2.49, −0.03]) predicted a lower probability.

### 3.3 Prediction of Confrontation Rupture Frequency

The results showed that interpersonal problems were also relevant for predicting confrontation ruptures, though the effects were generally weaker than for withdrawal ruptures (see Table 3). The overall interpersonal dysfunction model provided the best predictive performance (Δ ELPD = 0.0, SE = 0.0), followed by the quadrant model (Δ ELPD = −6.5, SE = 3.5), the dimension model (Δ ELPD = −7.7, SE = 4.7) and the octant model, which performed the worst (Δ ELPD = −13.8, SE = 5.5). ICCs ranged from 59.6% to 66.0%, again suggesting that confrontation ruptures were influenced by dyadic processes rather than solely individual patient characteristics.

**TABLE 3:** Prediction of confrontation rupture frequency.

	Model parameters		ICC	Model comparison	
Predictors of the models	Estimate (estimate error)	95% CI		Δ ELPD	SE (Δ ELPD)
Overall interpersonal dysfunction			59.59%	0.0	0.0
IIP mean	**−0.5063 (0.2359)**	**[−0.9904, −0.0656]**			
Interpersonal dimensions			59.73%	−7.7	4.7
Affiliation	−0.0706 (0.3223)	[−0.6935, 0.5737]			
Dominance	−0.0664 (0.3535)	[−0.7859, 0.6009]			
Interpersonal quadrants			60.77%	−6.5	3.5
Friendly–Dominant	0.2105 (0.1519)	[−0.0833, 0.5175]			
Friendly–Submissive	−0.3222 (0.1894)	[−0.7076, 0.0396]			
Hostile–Dominant	−0.2809 (0.1663)	[−0.6210, 0.0311]			
Hostile–Submissive	0.1636 (0.1423)	[−0.1101, 0.4521]			
Interpersonal octants			65.98%	−13.8	5.5
Domineering (PA)	0.1296 (0.2467)	[−0.3538, 0.6183]			
Self‐centred (bc)	−0.3479 (0.2162)	[−0.7962, 0.0529]			
Cold (DE)	0.0933 (0.3776)	[−0.6799, 0.8164]			
Socially inhibited (FG)	0.1544 (0.3487)	[−0.4886, 0.8753]			
Non‐assertive (HI)	−0.3219 (0.3485)	[−1.0237, 0.3454]			
Overly accommodating (JK)	−0.1488 (0.2503)	[−0.6750, 0.3185]			
Self‐sacrificing (LM)	**−0.6459 (0.4629)**	**[−1.3455, −0.0086]**			
Intrusive (NO)	0.5972 (0.3745)	[−0.0871, 1.3900]			

*Note:* Values in boldface highlight estimates with a credible departure from the null value.

Abbreviations: ICC, intraclass correlation coefficient; IIP, Inventory of Interpersonal Problems; SE, standard error; Δ ELPD, difference in Expected Log Predictive Density between models.

Higher overall interpersonal dysfunction predicted fewer confrontation ruptures (*β* = −0.506, 95% CI [−0.990, −0.066]). At the octant level, only self‐sacrificing tendencies significantly predicted fewer confrontation ruptures (*β* = −0.646, 95% CI [−1.346, −0.009]).

For the hurdle component, none of the predictors reached statistical significance, indicating that the likelihood of experiencing at least one confrontation rupture was not reliably associated with interpersonal problems characteristics.

## 4 Discussion

This study aimed to examine the frequency of alliance ruptures in the first three sessions of cognitive–behavioural therapy and investigated how interpersonal behaviours, as assessed by the overall severity, interpersonal dimensions (affiliation and dominance), quadrants and octants of the IIPs, predict rupture frequency. Our results show that rupture occurrences are common early in therapy, with withdrawal ruptures being coded more frequently than confrontation ruptures. Across models, interpersonal problems predicted the frequency of both withdrawal and confrontation ruptures, though the effects were stronger for withdrawal ruptures. The model with the interpersonal quadrants as predictors provided the best predictive performance for withdrawal ruptures, while overall interpersonal dysfunction was the strongest predictor of confrontation ruptures. For withdrawal ruptures, higher affiliation was associated with fewer occurrences of withdrawal ruptures. At the quadrant level, hostile–submissive tendencies were associated with more withdrawal ruptures, whereas friendly–submissive tendencies predicted fewer. More specific interpersonal problems also played a role: socially inhibited and non‐assertive tendencies were linked to increased withdrawal rupture frequency, while overly accommodating and cold interpersonal problem tendencies predicted fewer withdrawal ruptures. For confrontation ruptures, only the overall interpersonal dysfunction and problematic self‐sacrificing tendencies significantly predicted lower rupture frequency. The high ICCs suggest that rupture frequency was strongly influenced by therapist–patient dyads rather than individual patient characteristics alone.

Consistent with previous research, withdrawal ruptures were more commonly observed than confrontation ruptures (e.g., Cirasola et al. 2024; Lipsitz‐Odess et al. 2022). This finding is particularly relevant given the characteristics of the present sample, which consisted primarily of patients experiencing depressive or anxiety disorders. These internalizing disorders may be a reason that patients in situations of momentary interpersonal misattunements, rather than expressing their tension outwardly in a confrontational manner, may be more inclined to retreat and disengage from the dyad (Cirasola et al. 2024). Symptoms of these internalizing disorders, like social withdrawal, general avoidance and reduced emotional expression, can closely mirror the behaviours associated with withdrawal ruptures. Unlike confrontation ruptures, which involve overt expression of disagreement, withdrawal ruptures can be very subtle (Eubanks, Muran, and Safran 2014; Eubanks and Muran 2023). This overlap creates a critical dilemma: the patient's disengagement may be interpreted as part of their psychopathological syndrome rather than as a signal of rupture in the therapeutic dyad. Hence, the risk is that therapists may inadvertently attribute withdrawal behaviours to the patient's condition, overlooking the possibility that the withdrawal reflects a breakdown in the therapeutic alliance. As a result, the rupture may go undetected and unaddressed, leading to a further deterioration in collaboration and hindering the patient's therapeutic progress (Rubel et al. 2018). Consequently, it would be invaluable for psychotherapists to have additional resources or information that may help them recognize or be alert to these alliance ruptures. One potential route for this is through the understanding of the patient's interpersonal problems (Gómez Penedo et al. 2024; Zilcha‐Mano and Muran 2024). This study examined whether specific types of interpersonal problems predict rupture frequencies and which level of interpersonal assessment provides the most clinically relevant insights into alliance ruptures in the present sample.

Generally, our results indicate that specific interpersonal problems were significantly associated with the frequency of withdrawal ruptures. We had hypothesized that overall interpersonal dysfunction would predict greater withdrawal rupture frequency (H1), as higher levels of interpersonal distress were expected to contribute to heightened relational strain and misattunements characterized by withdrawal behaviour within the dyad. However, this was not supported, suggesting that the global measure of interpersonal dysfunction may not be well‐suited to capturing specific rupture behaviours within a dyad. Withdrawal ruptures likely emerge from distinct interpersonal dynamics rather than general distress alone. Instead, higher affiliation significantly predicted fewer withdrawal ruptures, aligning with our expectations and previous research indicating that higher affiliative tendencies facilitate the formation of stronger alliances (Hardy et al. 2001; Muran et al. 1994). In contrast, dominance did not significantly predict withdrawal ruptures, suggesting that this dimension alone may not sufficiently capture the interpersonal dynamics underlying withdrawal behaviours. Given that dominance reflects a more active stance in interpersonal interactions, it may be less relevant for understanding a passive rupture pattern such as withdrawal. The quadrant model, which integrates both affiliation and dominance into distinct interpersonal styles, provided the best predictive performance. This suggests that considering interpersonal tendencies as combinations of both dimensions, rather than examining them separately, offers a more precise understanding of how withdrawal ruptures unfold. At the quadrant level of the IIPs, friendly–submissive tendencies of the patient before therapy predicted fewer withdrawal alliance ruptures throughout the first three therapy sessions, while hostile–submissive tendencies predicted significantly more withdrawal ruptures. This is consistent with previous research that suggested that friendly submissiveness might be beneficial for the therapeutic alliance (Gurtman 1996; Muran et al. 1994). This association may reflect the tendency of friendly submissive individuals to prioritize harmony and avoid conflict, even in the face of momentary interpersonal misattunement. These patients may be less likely to withdraw from the therapeutic dyad because their primary goal is to maintain a positive relationship with the therapist. In contrast, hostile–submissive tendencies predicted significantly more withdrawal ruptures across the first sessions, which was expected (H5). It indicates that patients with hostile–submissive tendencies may internalize their frustration during moments of interpersonal tensions in therapy sessions, leading to withdrawal from the dyad and the therapeutic process itself. Rather than confronting the therapist, these patients show forms of passive resistance away from their therapist. Interestingly, higher affiliative and friendly–submissive tendencies increased the likelihood of experiencing at least one withdrawal rupture (hurdle component) but predicted fewer withdrawal ruptures once they occurred. In contrast, higher hostile–submissive scores were linked to a lower probability but higher frequency when they did occur. This suggests that while some interpersonal problem tendencies may make patients more prone to initial rupture episodes, the burden of interpersonal strain may shape how frequently ruptures recur throughout therapy. Lastly, the exploration on the octant level revealed that socially inhibited and non‐assertive patients showed significantly more withdrawal ruptures, whereas problems with cold and overly accommodating tendencies predicted fewer withdrawal ruptures. Social inhibition and non‐assertiveness often involves the discomfort in and avoidance of interpersonal situations (Ruiz et al. 2004; Thomas, Brähler, and Strauß 2011). This pattern of avoidance seemingly complicates the therapeutic alliance. Interestingly, neither friendly–dominant nor hostile–dominant behaviour tendencies significantly predicted subsequent ruptures of patient's withdrawal. In line with theory and previous research, this is not surprising, as dominant interpersonal tendencies are generally not associated with the passive nature of withdrawal ruptures (Luo et al. 2021; Muran et al. 1994).

For confrontation ruptures, the overall interpersonal dysfunction model demonstrated the best predictive performance. However, contrary to our hypothesis, higher interpersonal dysfunction predicted fewer confrontation ruptures rather than more. The most apparent explanation for this finding is that, in the present sample, problematic interpersonal tendencies primarily reflected excessive submissiveness rather than dominant interpersonal behaviours. Given that confrontation ruptures involve a direct and outward expression of dissatisfaction (Eubanks and Muran 2023), it is plausible that the overall severity of interpersonal distress in this sample did not manifest in assertive or dominant ways that would typically contribute to confrontation ruptures. Even though the overall interpersonal dysfunction model demonstrated the best predictive performance, its broad nature may limit its utility in identifying specific problematic interpersonal styles that contribute to confrontation ruptures. Moreover, it is very important to interpret these results cautiously, as the low overall frequency of confrontation ruptures in the sample likely reduced statistical power. Although the octant model demonstrated the weakest predictive performance overall, it did reveal that higher levels of self‐sacrificing tendencies predicted fewer confrontation ruptures. This aligns with theoretical expectations, as individuals with problematic self‐sacrificing tendencies are often excessively focused on pleasing others (Alden, Wiggins, and Pincus 1990; Ruiz et al. 2004; Thomas, Brähler, and Strauß 2011). As a result, they may be less likely to engage in confrontational behaviours themselves or elicit confrontation from their therapists. Future research with larger samples and more diverse interpersonal problem profiles is needed to clarify these nuances.

## 5 Limitations

There are several limitations that should be considered when interpreting the presented results. First, as mentioned previously, the sample may have been underpowered, particularly given the lower frequency of confrontation ruptures. While prior research suggests that withdrawal ruptures are generally more frequent than confrontation ruptures, it remains possible that confrontation ruptures were undercoded in this study. If so, this may have led to an underestimation of their prevalence and impact, limiting statistical power to detect certain effects. Second, the operationalization of rupture frequency in five‐minute segments introduces some limitations. Specifically, the coding system identified whether a rupture occurred within each segment but did not quantify the exact number of rupture events within that timeframe. While this serves as a reasonable proxy for rupture frequency, it remains possible that multiple rupture events occurred within a single segment or that a rupture extended across segment boundaries. However, the latter scenario was explicitly instructed to be noted by coders, and no such instances were recorded. Thus, while the segmentation approach provides a structured way to assess rupture frequency, it may still underestimate the true number of rupture events. Third, rupture‐resolution theory (Eubanks, Muran, and Safran 2019; Safran et al. 2001; Safran and Muran 1996) emphasizes not only rupture occurrences but also the effectiveness of repair strategies in shaping the therapeutic alliance. However, because repair attempts were very rare in our data, we were unable to examine their role. Future research should explore the interplay between ruptures and repairs to better understand their combined impact on alliance development. Fourth, despite the dyadic nature of ruptures, our analyses were limited to patient‐specific predictors. Therapist characteristics were not available, yet a complementary assessment of therapist behaviours and interpersonal styles could provide valuable insights into the interactive nature of rupture processes. Fifth, the IIP‐32 relies on self‐report, which may be influenced by social desirability or limited self‐awareness on the part of the patients. Patients may underreport certain interpersonal behaviours, particularly those that are more hostile or aggressive, which could introduce biases into the data. Lastly, while our sample was naturalistic, it primarily consisted of patients with internalizing disorders, such as depression and anxiety. This may limit the generalizability of our findings to other populations, particularly those with externalizing disorders or more overtly dominant or confrontational tendencies.

## 6 Conclusion

In conclusion, the findings of our study highlight the significant role of problematic interpersonal tendencies in predicting alliance ruptures in the first three sessions of cognitive–behavioural therapy. Specifically, withdrawal ruptures were associated with submissive interpersonal tendencies, while confrontation ruptures were less frequent and less systematically related to patient‐reported interpersonal dysfunction. These results suggest that therapists may benefit from attending not only to broad interpersonal distress but also to specific interpersonal styles that shape the emergence and frequency of rupture behaviours. By becoming more attuned to these dynamics, therapists may be better equipped to recognize and address alliance ruptures, even when they manifest in subtle or passive forms, thereby fostering stronger therapeutic collaboration.

## Ethics Statement

All subjects gave their written consent before their data was analysed. The consent process was in line with ethical standards and ensured that patients were fully informed about the potential use of their data in scientific studies. This study was conducted in accordance with the Declaration of Helsinki.

## Consent

All patients provided informed consent as part of their routine outcome monitoring procedures, agreeing that their data could be used for research purposes.

## Conflicts of Interest

The authors declare no conflicts of interest.

## Data Availability

The data analyzed in this study are not publicly available due to the need to protect patient privacy and confidentiality.
